# Heparan Sulfate Proteoglycan Clustering in Wnt Signaling and Dispersal

**DOI:** 10.3389/fcell.2020.00631

**Published:** 2020-07-14

**Authors:** Yusuke Mii, Shinji Takada

**Affiliations:** ^1^National Institute for Basic Biology, National Institutes of Natural Sciences, Okazaki, Japan; ^2^Exploratory Research Center on Life and Living Systems, National Institutes of Natural Sciences, Okazaki, Japan; ^3^Department of Basic Biology, Graduate University for Advanced Studies (SOKENDAI), Okazaki, Japan; ^4^Japan Science and Technology Agency, PRESTO, Saitama, Japan

**Keywords:** HSPG, glypican, HS cluster, *N*-sulfation, NDST, Wnt, signaling, morphogen gradient

## Abstract

Wnt, a family of secreted signal proteins, serves diverse functions in animal development, stem cell systems, and carcinogenesis. Although Wnt is generally considered a morphogen, the mechanism by which Wnt ligands disperse is still debated. Heparan sulfate proteoglycans (HSPGs) are extracellular regulators involved in Wnt ligand dispersal. *Drosophila* genetics have revealed that HSPGs participate in accumulation and transport of Wnt ligands. Based on these findings, a “restricted diffusion” model, in which Wnt ligands are gradually transferred by repetitive binding and dissociation to HSPGs, has been proposed. Nonetheless, we recently found that HSPGs are not uniformly distributed, but are locally clustered on cell surfaces in *Xenopus* embryos. HSPGs with *N*-sulfo-rich HS chains and those with *N*-acetyl-rich unmodified HS chains form different clusters. Furthermore, endogenous Wnt8 ligands are discretely accumulated in a punctate fashion, colocalized with the *N*-sulfo-rich clusters. Based on these lines of evidence, here we reconsider the classical view of morphogen spreading controlled by HSPGs.

## Introduction

It is generally recognized that functions of secreted signaling proteins, or morphogens, are affected by heparan sulfate proteoglycans (HSPGs). Some of the greatest contributions to this view have come from genetic research involving *Drosophila* (Yan and Lin, 2009). Historically, genes responsible for segment polarity cuticle phenotypes, which are similar to the phenotypes of *wingless* (*wg; Drosophila* ortholog of *Wnt1*), and *hedgehog* mutant embryos, were identified as genes involved in glycogenesis of heparan sulfate (HS) chains (Binari et al., 1997; Hacker et al., 1997; Haerry et al., 1997; Lin and Perrimon, 1999). Furthermore, genes encoding core proteins of HSPGs, such as glypicans (Filmus et al., 2008) and enzymes for glycosaminoglycan (GAG) chains, such as EXTs (Busse-Wicher et al., 2014), regulate signal transduction and extracellular trafficking of morphogen proteins. In parallel with genetic research, biochemical studies have shown that HS chains have high affinity for many kinds of morphogens, including FGF (Esko and Selleck, 2002) and Wnt (Gao et al., 2016). Based on these studies, interactions between morphogens and HSPGs have been considered crucial for generation and maintenance of signaling (Sarrazin et al., 2011).

Interestingly, HSPGs show variable GAG chain composition and core protein structure. GAG chains are especially highly modified by *N*- and *O*-sulfation and the extent of these modifications is variable (Esko and Selleck, 2002). This variation has been recognized for a long time, but it was unclear whether modifications of individual HS chains vary within individual cells. However, our recent studies have revealed that *N*-sulfo-rich and *N*-acetyl-rich HSPGs form different clusters on individual cells (Mii et al., 2017). This new finding suggests that distinct clusters of HSPGs regulate Wnt signaling differently and that distribution of these clusters may govern dispersal of signaling proteins and may define the signaling range (distance) of morphogens. In this review, we will first provide a general understanding of the structural and functional diversity of HSPGs. Then we will document the clustering of specifically modified HSPGs and propose a model by which Wnt signaling is governed via interaction with clusters of HSPGs.

## Wnt Signaling and Intercellular Delivery

Wnt ligands activate several distinct cellular signaling pathways, including the Wnt/β-catenin and Wnt/JNK pathways (Niehrs, 2012). Upon activation of the Wnt/β-catenin pathway, Wnt ligands promote assembly of signaling complexes called “signalosomes,” which involve Frizzled (Fz) receptors, Lrp5/6 coreceptors, and cytoplasmic components Dishevelled (Dvl) and Axin (Bilic et al., 2007; Kikuchi et al., 2009). Formation of signalosomes results in stabilization of cytosolic β-catenin, thereby activating Tcf transcription factors and their target genes (Kikuchi et al., 2009). In contrast, the Wnt/JNK pathway requires Fz receptors and Dvl, and it activates small GTPases, such as Rho and Rac, and the protein kinase, JNK (Niehrs, 2012). In humans and mice, 19 Wnt ligands have been identified, and some of them, Wnt1, Wnt3a, and Wnt8, preferentially activate Wnt/β-catenin whereas others, Wnt5a and Wnt11, activate mainly the Wnt/JNK pathway.

Most Wnt ligands are modified with palmitoleic acid and delivered to neighboring cells (Takada et al., 2006). A number of mechanisms have been proposed to explain Wnt delivery (Takada et al., 2017). For instance, extracellular vesicles, like exosomes (Gross et al., 2012), lipoprotein particles (Panakova et al., 2005), and filopodia-like cellular processes called cytonemes (Stanganello et al., 2015; Stanganello and Scholpp, 2016) have been shown to mediate Wnt delivery. In contrast, secreted Wnt does not appear to exist as a monomer, because no monomeric form was detected in the culture medium of Wnt3a-expressing mouse L cells (Takada et al., 2018). Rather, Wnt3a protein forms heteromeric complexes with partner proteins or assembles itself into high-molecular-weight complexes, which are less diffusible and which easily dissociate to form complexes with Fz receptors (Takada et al., 2018). In embryos, some Wnt-binding proteins facilitate Wnt delivery. Some secreted Frizzled-related proteins (sFRP), sFRP2 and Frzb, form heteromeric complexes with Wnt so as to enhance their delivery in *Xenopus* embryos (Mii and Taira, 2009; Takada et al., 2018). Similarly, swim, a member of the Lipocalin family of extracellular transport proteins, facilitates Wg diffusion in *Drosophila* imaginal disks (Mulligan et al., 2012).

In addition to these delivery systems, HSPGs are also involved in Wnt delivery. Genetic studies in *Drosophila* suggest that HSPGs either enhance signaling by Wnt ligands or delivery of Wnt ligands to neighboring cells in a context-dependent manner (Han et al., 2004b; Franch-Marro et al., 2005; Yan and Lin, 2009). It has been proposed that HSPGs mediate Wnt delivery by a restricted-diffusion mechanism, in which Wnt ligands are transported in a “bucket brigade” manner by repeated association and dissociation with HSPGs on cell membranes (Yan and Lin, 2009). The restricted diffusion model has been adopted to explain the mechanism of delivery of several secreted signal proteins, including Wnt. However, results of recent quantitative analyses do not appear to support this model. For instance, Dpp diffuses freely in *Drosophila* wing disks (Zhou et al., 2012). Similarly, freely diffusing forms of Wnt8 have also been detected in *Xenopus* embryos (Mii et al., 2020). These examples imply that the restricted diffusion model should be carefully reconsidered.

## Characteristics of Core Proteins and Sugar Chains of HSPGs

HSPGs are composed of core proteins with attached heparan sulfate (HS) GAG chains. Approximately 20 core proteins have been identified and are classified into several families, based upon their structures (Sarrazin et al., 2011). Proteins of the two major families, the glypican and syndecan families, are attached to cell membranes (Bernfield et al., 1999). Glypican family proteins, including GPC1-6 in vertebrates and Dally and Dally like protein (Dlp) in *Drosophila*, are linked to the membrane by glycerophosphatidylinositide (GPI)-anchors. These glypicans can be divided into two subgroups based upon amino acid sequence homology. GPC1/2/4/6 and Dlp form one group, while GPC3/5 and Dally form the other (Filmus et al., 2008). Evidence suggests some functional differences among glypicans, but it remains to be seen whether such differences result from structural differences between the subfamilies (Han et al., 2004b; Franch-Marro et al., 2005; Yan and Lin, 2009). Glypican family proteins commonly have a cysteine-rich domain at their N-termini and several HS attachment sites close to the membrane anchoring site. Interestingly, the structure of this cysteine-rich domain is similar to that of Fz and it mediates Wnt binding (Topczewski et al., 2001). On the other hand, syndecan family members (SDC1-4 in vertebrates and a single syndecan in *Drosophila*) are transmembrane proteins. Syndecans bear HS chains at their N-termini and some SDCs also bear chondroitin sulfate (Gondelaud and Ricard-Blum, 2019). In addition to these two types of cell surface HSPGs, secreted HSPGs (perlcan, agrin, and collagen type XVIII in vertebrates and terribly reduced optic lobes (trol) in *Drosophila*), have also been identified. Secreted HSPGs are mainly found in the extracellular matrix (Sarrazin et al., 2011).

**FIGURE 1:**
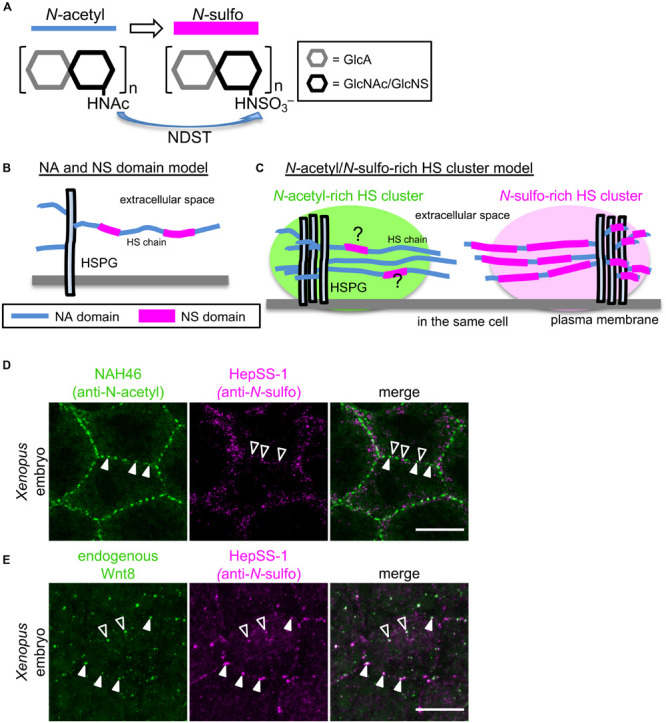
Models explaining the diversity of HS chain modification of HSPGs. **(A)** *N*-sulfation of GlcA-GlcNAc units of HS chains by NDST. HS chains are synthesized by sequential actions of glycosyl transferases and modification enzymes. After polymerization of disaccharide units, elongated HS chains are extensively modified by sulfotransferases, including GlcNAc *N*-deacetylase/*N*-sulfotransferase (NDST), which catalyzes *N*-sulfation of GlcA-GlcNAc units of HS chains. **(B)** NA and NS domain model. Analysis of the oligosaccharides of HS chains obtained by digestion under conditions in which *N*-sulfated GlcA-GlcNAc units are selectively attacked, showed that various heparan sulfate samples all contained regions of consecutive *N*-sulfated GlcA-GlcNAc units, as well as contiguous *N*-acetylated ones (Gallagher and Walker, 1985; Maccarana et al., 1996; Bernfield et al., 1999). These findings suggest that modifications occur in clusters of variable length (*N*-sulfated or NS domains), which are interspersed among unmodified domains (*N*-acetylated or NA domains). It has been proposed that these two domains coexist on single HS chains. **(C)** *N*-acetyl-rich and *N*-sulfo-rich HS cluster model. Recently, Mii et al. found that *N*-sulfo-rich and *N*-acetyl-rich HSPGs are clustered independently on cell membranes of *Xenopus* embryos and on cultured cells (Mii et al., 2017). This new finding strongly suggests that NS and NA domains do not exist randomly on individual HS chains. Rather, the extent of *N*-sulfation appears to vary between HSPG clusters. Although *N*-sulfo-rich and *N*-acetyl-rich clusters rarely overlap on the cell surface, it cannot be excluded that HS chains in *N*-sulfo-rich and *N*-acetyl-rich HS clusters may contain some NA and NS domains, respectively. **(D)** *N*-acetyl-rich and *N*-sulfo-rich HS clusters in a *Xenopus* embryo. Double color immunostaining with direct-labeled NAH46 (anti-*N*-acetyl subunits) and HepSS-1 (anti-*N*-sulfo subunits) antibodies shows clustered distributions of HS chains recognized by these antibodies (Mii et al., 2017). Notably, NAH46 and HepSS-1 staining do not largely overlap, but rather show distinct distributions. **(E)** Endogenous Wnt8 colocalized with *N*-sulfo rich HS clusters. Double color immunostaining with anti-Wnt8 and HepSS-1 antibodies shows that Wnt8 staining mostly overlaps with HepSS-1 staining. Colocalization is indicated with closed (cell boundary) and open (inside cells) arrowheads. Scale bars, 20μm.

HS chains are linear polysaccharides that contain 20–150 repeating disaccharide units of *N*-acetylglucosamine (GlcNAc) and either uronic acid [glucuronic acid (GlcA) or iduronic acid (IdoA)] (Sarrazin et al., 2011). These chains are synthesized in the Golgi by sequential actions of glycosyl transferases and modification enzymes (Esko and Selleck, 2002). HS chain synthesis is initiated by adding tetrasaccharide linkers to serine residues in the core proteins. Then, a number of disaccharide units are sequentially attached to HS chains by co-polymerases known as Ext1 and Ext2 [*Tout-velu* (*Ttv*) and *Sister of ttv* (*Sotv*) in *Drosophila*]. Following this polymerization process, elongated HS chains are extensively modified by sulfotransferases and an epimerase. For instance, GlcNAc *N*-deacetylase/*N*-sulfotransferase [NDST1-4, or *sulfateless* (*sfl*) in *Drosophila*] catalyzes GlcNAc *N*-deacetylation and *N*-sulfation (Figure 1A), and C5 epimerase converts GlcA to IdoA. In addition, 2-*O*-Sulfotransferase, 6-*O*-Sulfotransferases, and 3-*O*-Sulfotransferases variously catalyze *O*-sulfation at C2 of uronic acid, at C6 of *N*-acetyl- and *N*-sulfo-glucosamine, and at C3 of glucosamine, respectively. Notably, these reactions do not proceed to completion in the Golgi, resulting in structural diversity of HS chains. Domains rich in *N-*sulfated disaccharides and those rich in unmodified disaccharides, that is, *N-*acetyl disaccharides, exist on HS chains (NS or NA domains, respectively, Figure 1B; Gallagher and Walker, 1985; Maccarana et al., 1996; Bernfield et al., 1999). In the *N-*sulfated rich domain, *O-*sulfation is also frequently detected. Thus, these modifications appear to occur commonly among adjacent disaccharides in HS synthesis.

## Involvement of Glypicans in Wnt Signaling and Distribution

Genetic studies using *Drosophila* illustrate the importance of HSPGs in Wnt signaling. For instance, loss-of-function of glypican, Dally or Dlp, results in reduction of Wg signaling and extracellular Wg levels in wing disks (Franch-Marro et al., 2005; Han et al., 2005). Similarly, Wg signaling and the extracellular distribution of Wg are reduced in cells deficient in genes required for biosynthesis of HS chains, including *sugarless* (UDP-glucose dehydrogenase) (Hacker et al., 1997; Haerry et al., 1997), *sfl* (NDST) (Lin and Perrimon, 1999; Baeg et al., 2004), and *Ttv* and *Sotv* (EXTs) (Han et al., 2004a; Takei et al., 2004). Thus, HSPGs are essential for proper signaling and distribution in fly development.

In vertebrates, HSPGs may modulate various extracellular signaling proteins, but several lines of evidence confirm their involvement in Wnt signaling. For instance, in zebrafish and *Xenopus*, disruption of *gpc4/knypek* function causes defects in convergent extension movement, which is modulated by Wnt/JNK signaling, during gastrulation (Topczewski et al., 2001; Ohkawara et al., 2003). Mouse embryos lacking *Gpc3* show reduced Wnt/JNK signaling (Song et al., 2005). Cell culture studies indicate that glypicans appear to modulate β-catenin-dependent and -independent pathways in vertebrate cells, depending on different membrane microdomains (Sakane et al., 2012). In addition to glypicans, other HSPG core proteins, syndecan and perlecan, are involved in Wnt signaling, but will not be considered here.

One of the important issues regarding Wnt binding is whether HSPG core proteins or GAG chains are required. Since Gpc3 lacking GAG chains can bind to several Wnt ligands and can positively regulate canonical Wnt signaling (Capurro et al., 2005), glypican core protein appears sufficient for Wnt binding. In contrast, involvement of GAG chains for interaction with Wnt has also been reported. Wnt8 accumulation upon overexpression of Gpc4 or Gpc5 appears to be HS chain-dependent, because ΔGAG mutants of these glypicans do not accumulate Wnt8 (Mii et al., 2017). Furthermore, *Drosophila* mutants with impaired HS chains suggest essential roles for HS chains in Wg binding and regulation (Lin and Perrimon, 1999; Baeg et al., 2004; Takei et al., 2004). Thus, in addition to core protein, HS chains appear to be required for Wnt signaling *in vivo.*

## HS Clusters and Wnt Signaling

### Assembly of HSPGs With Similarly Modified HS Chains

For better understanding of HSPG-mediated Wnt signaling and dispersal, it is important to understand the spatial distribution of HSPGs in tissues or cells. Given the variability in core proteins and HS chain composition, it is important to examine the expression pattern of each core protein and fine localization patterns of HS chain modifications. Using two monoclonal antibodies, HepSS-1 (Kure and Yoshie, 1986; van den Born et al., 2005) and NAH46 (Suzuki et al., 2008), which recognize HS chains of *N*-sulfated (GlcA-GlcNS)_n_ and unmodified *N*-acetylated (GlcA-GlcNAc)_n_ structures, respectively, distributions of differently modified HS chains were examined in *Xenopus* embryos at gastrula stage. At this stage, Wnt8 is expressed in the ventral and lateral marginal zone and participates in ventral mesodermal patterning. Immunostaining with either of these two antibodies showed that HSPGs that react with these antibodies are not uniformly distributed on cell surfaces. Instead, they aggregate locally to form discrete clusters (Figures 1C,D). Increased or decreased expression of NDST1, which catalyzes *N-*sulfation of HS disaccharides, showed that HepSS-1 and NAH46 clusters actually represent *N-*sulfo- or *N-*acetyl-rich clusters, respectively. These results suggest that HSPGs with *N-*sulfo-rich or *N-*acetyl-rich HS chains form discrete clusters, designated “HS clusters” on the cell surface (Mii et al., 2017).

### Distinct Roles of HS Clusters in Wnt Signaling and Dispersal

These two types of clusters show different specificities for secreted signal proteins. First, Wnt ligands are specifically colocalized with *N*-sulfo-rich clusters. In *Xenopus* embryos, endogenous, as well as overexpressed Wnt8 ligands, are also distributed in a punctate pattern on cell surfaces. Most of these dots overlap with *N*-sulfo-rich clusters (Figure 1E). This interaction between Wnt8 and *N-*sulfo-rich HSs is dependent on *N-*sulfation, because overexpression of *ndst1* increases *N-*sulfation levels and Wnt8 accumulation, and vice versa. Thus, *N-*sulfo-rich HS clusters serve as major scaffolds where Wnt8 ligands are trapped in *Xenopus* embryos (Mii et al., 2017). On the other hand, *N-*acetyl-rich HS clusters serve as scaffolds for Frzb (Leyns et al., 1997; Wang et al., 1997), a member of the secreted Frizzled-related protein (sFRP) family (Bovolenta et al., 2008; Mii and Taira, 2011).

Wnt ligands trigger formation of signalosomes (Bilic et al., 2007), which are subsequently internalized by caveolin-mediated endocytosis (Yamamoto et al., 2006; Kikuchi et al., 2009). In signalosomes, Fz receptors, LRP5/6 coreceptors, and cytoplasmic components, including Dvl and Axin, are assembled to facilitate phosphorylation of LRP5/6, which is essential for activation of canonical Wnt signaling (Bilic et al., 2007; Kikuchi et al., 2009). Notably, *N-*sulfo-rich HS clusters, but not *N-*acetyl-rich HS clusters, are frequently internalized (Mii et al., 2017). In the presence of Wnt8, phosphorylated LRP6 is preferentially detected at *N-*sulfo-rich HS clusters, but NDST1 knockdown reduces LRP6 phosphorylation and also Wnt/β-catenin signaling. Consistent with these results, NDST1 knockdown inhibits secondary axis formation caused by ventral injection of *wnt8* mRNA in *Xenopus* embryos. It was also shown that Wnt3a and signalosome components are localized with *N*-sulfo-rich HS in HeLa cells. These results suggest that *N-*sulfo-rich HS clusters are required for Wnt/β-catenin signaling and signalosome formation in *Xenopus* embryos and cultured cells. Because *N-*sulfo-rich HS clusters form independently of Wnt ligand, it seems probable that *N-*sulfo-rich HS clusters serve as pre-existing scaffolds to assemble signalosomes (Mii et al., 2017).

As described above, Wnt8 associates with *N-*sulfo-rich HS clusters in *Xenopus* embryos. However, when Frzb is overexpressed, Wnt8 association with *N*-sulfo-rich HS clusters decreases, and Wnt8 then associates with *N*-acetyl-rich clusters via Frzb (Mii et al., 2017). Given that Wnt8 forms a heteromeric complex with Frzb (Leyns et al., 1997; Wang et al., 1997), Wnt8 that forms these heteromeric complexes probably associates with *N*-acetyl-rich clusters. In contrast, other forms of Wnt8, such as Wnt8 in extracellular vesicles or in homomeric complexes (Takada et al., 2018), as indicated above, may associate with *N*-sulfo-rich clusters. In *Xenopus* embryos, ectopically expressed Wnt8 shows only a short distribution range, but this range can be expanded if Frzb, which shows a much longer one, is coexpressed with Wnt8 (Mii and Taira, 2009). Thus, in this context, it seems probable that *N*-acetyl-rich HS clusters serve as scaffolds that enable more long-range delivery of Wnt8/Frzb complexes.

**FIGURE 2:**
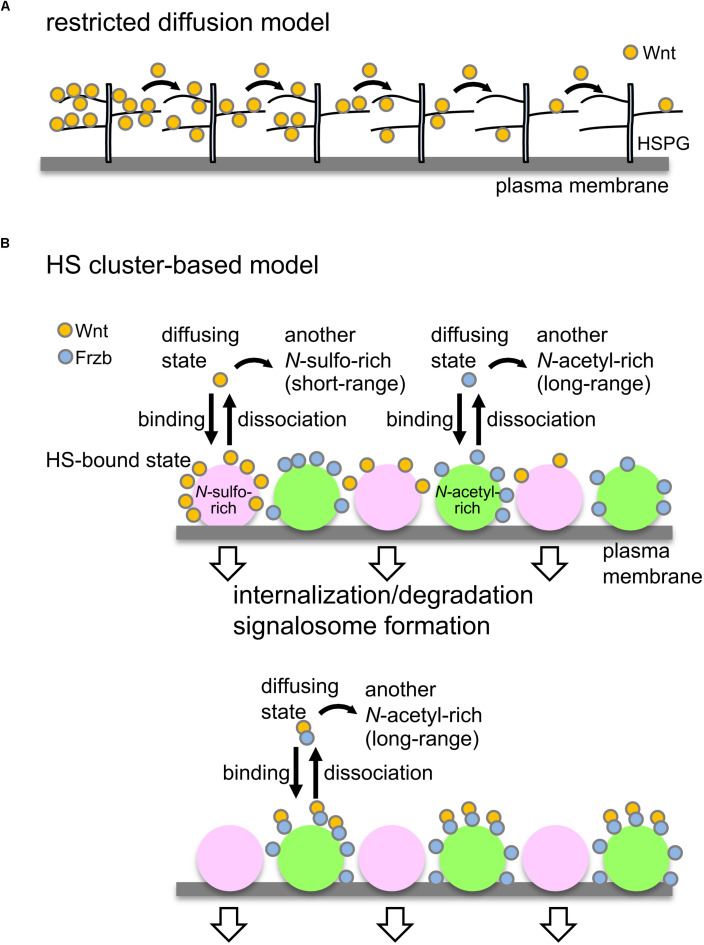
Models to control Wnt signaling and dispersal by HSPGs. **(A)** Restricted diffusion model. Based on genetic studies in *Drosophila*, it has been proposed that HSPGs mediate Wnt delivery by a restricted-diffusion mechanism, in which Wnt ligands are transported in a “bucket brigade” manner by repeated association and dissociation with HSPGs on cell membranes (Yan and Lin, 2009). **(B)** Model to explain Wnt signaling and delivery by clustering of HSPGs. Wnt8 preferentially binds to *N*-sulfo-rich HS clusters and Frzb binds to *N*-acetyl-rich clusters (upper; Mii et al., 2017). Accumulation of Wnt8 on *N*-sulfo-rich HS clusters leads to signalosome formation and internalization of Wnt8, which may contribute to degradation of Wnt8. When Frzb is abundant (lower), Wnt8-Frzb complexes bind to *N*-acetyl-rich HS clusters, which may reduce degradation of Wnt8 (Mii et al., 2017). Given that these two clusters are not distributed uniformly on the cell surface, it seems unlikely that Wnt ligands are transported in a “bucket brigade” manner between these clusters. Since *N*-sulfo-rich HS clusters are frequently internalized, this cluster appears to shorten the distribution range of Wnt8 (Mii and Taira, 2009). On the other hand, *N*-acetyl-rich HS clusters tend to remain on the cell surface, resulting in long-range distributions of Frzb as well as Wnt8-Frzb complexes (Mii and Taira, 2009). One possible model is that the balance of Wnt interactions between *N*-sulfo-rich HS clusters and *N*-acetyl-rich HS clusters may regulate Wnt signaling range in tissues.

We recently found that some Wnt8 diffuses freely, but that the majority of it is bound to cell surface scaffolds, probably HSPG clusters (Mii et al., 2020). Interestingly, Wnt8 molecules bound to scaffolds seem to be released occasionally, but diffusing away rather than being trapped on the adjacent cell surface. Thus, in contrast to predictions by the restricted diffusion model (Figure 2A), “bucket-brigade”-type transfer of Wnt8 was not detectable on cell surfaces (Mii et al., 2020). Given that scaffolds are scattered on cell membranes, Wnt8 molecules, probably associating with Frzb, are likely to be delivered over long distances by jumping between the scaffolds, probably provided by *N*-acetyl-rich clusters (Figure 2B; Mii and Taira, 2009; Mii et al., 2017).

### Specificity of Glypicans for Distinct HS Clusters

Evidence suggests that glypicans are the major core proteins of these HS clusters. PI-PLC treatment and cholesterol removal with methyl-β-cyclodextran reduced HS clusters, suggesting that GPI-anchored proteins, most probably glypicans, are involved in clustering. Among glypicans, Gpc4 and Gpc5 are highly expressed in *Xenopus* gastrulae. We demonstrated that Gpc5, an ortholog of *Drosophila* Dally, bears mainly *N*-sulfo-rich HS, whereas Gpc4, an ortholog of Dlp, bears both *N*-sulfo-rich and *N*-acetyl-rich HS (Mii et al., 2017). Thus, although glypican core proteins provide a molecular basis for clustering, composition of core proteins appears to differ between the two cluster types. On the other hand, it is still uncertain whether other core proteins, such as syndecans, are involved in formation of HS clusters.

Interestingly, Dally and Dlp appear to modulate Wg signaling and distribution differently in *Drosophila* wing disk (Franch-Marro et al., 2005; Han et al., 2005). Dally enhances Wg signaling through DFz2 receptors and internalization of receptor complexes. On the other hand, Dlp exhibits biphasic activity in Wg signaling and distribution. While Dlp acts as a positive regulator in regions distal from Wg-producing cells, it also acts as a negative regulator proximally. This biphasic behavior can be explained if Dlp delivers captured Wg to Fz receptors on the same cell or passes it to neighboring cells, depending on the cellular context. In view of phylogenetic relationships of these *Drosophila* glypicans to Gpc5 and Gpc4, as shown above, we propose that the specificity of the two glypican subfamilies in Wnt signaling and distribution is consistent among invertebrates and vertebrates.

### Mechanisms by Which Discrete HS Clusters Are Formed

It remains to be determined how these two distinct types of clusters are generated. To answer this question, understanding the regulation of HS modifications in the ER and/or the Golgi seems to hold the key. Interestingly, it has been suggested that NDST1 is associated with Ext1 or Ext2 in the Golgi, forming an HS biosynthesis complex called a GAGosome (Esko and Selleck, 2002). The stoichiometry and composition of these enzymes in GAGosomes may affect modifications of HS chains, such as *N*-sulfation (Presto et al., 2008). Given that some types of GAGosomes are localized in particular regions in the Golgi, this spatial heterogeneity may generate differential *N*-sulfation even within a single cell. Consistent with this idea, *sulfateless*, *Drosophila* NDST localizes in a specific sub-compartment of the Golgi apparatus (Yano et al., 2005). On the other hand, biosynthesis and transport of 3′-phosphoadenyl 5′-phosphosulfate (PAPS), a sulfuryl group donor, are required for proper sulfation reactions (Kurima et al., 1998; Esko and Selleck, 2002; Kamiyama et al., 2003). If local abundance or absence of PAPS exists in Golgi, this could be a mechanism generating distinct modifications of HS clusters.

## Perspectives

In this review, we proposed that novel types of HSPGs, *N*-sulfo- and *N*-acetyl-rich HS clusters, provide insight into regulation of secreted signaling proteins, such as Wnt. HS clusters enable cells to regulate Wnt8 and its binding protein, Frzb, in a controlled manner. Although organization of HSPGs is difficult to analyze by biochemical methods, we assume that various types of HS clusters could be involved in many aspects of embryogenesis and homeostasis. Hypothetical HS clusters with various modifications could serve as specific platforms on cell surfaces for various secreted proteins, as exemplified by combinations of Wnt8-*N*-sulfo-rich HS clusters and Frzb-*N*-acetyl-rich HS clusters (Mii et al., 2017). Future studies will focus on the generality of this finding, especially in other biological systems and with other modifications of HS chains. Given that HSPGs modulate Wnt signaling in various diseases (Capurro et al., 2005; Zittermann et al., 2010; Lund et al., 2020), HS modification and clustering could influence disease progression.

## Author Contributions

YM and ST conceived and wrote the review together, and approved the submitted version.

## Conflict of Interest

The authors declare that the research was conducted in the absence of any commercial or financial relationships that could be construed as a potential conflict of interest.
